# Towards a Proteomic Catalogue and Differential Annotation of Salivary Gland Proteins in Blood Fed Malaria Vector *Anopheles culicifacies* by Mass Spectrometry

**DOI:** 10.1371/journal.pone.0161870

**Published:** 2016-09-07

**Authors:** Ritu Rawal, Sonam Vijay, Kavita Kadian, Jagbir Singh, Veena Pande, Arun Sharma

**Affiliations:** 1 Protein Biochemistry and Structural Biology, National Institute of Malaria Research (ICMR), Sector-8, Dwarka, New Delhi, India; 2 Department of Biotechnology, Kumaun University, Nainital, Uttarakhand, 263001, India; Council for Scientific and Industrial Research, INDIA

## Abstract

In order to understand the importance of functional proteins in mosquito behavior, following blood meal, a baseline proteomic dataset is essential for providing insights into the physiology of blood feeding. Therefore, in this study as first step, in solution and 1-D electrophoresis digestion approach combined with tandem mass spectrometry (nano LC-MS/MS) and computational bioinformatics for data mining was used to prepare a baseline proteomic catalogue of salivary gland proteins of sugar fed *An*. *culicifacies* mosquitoes. A total of 106 proteins were identified and analyzed by SEQUEST algorithm against mosquito protein database from Uniprot/NCBI. Importantly, D7r1, D7r2, D7r4, salivary apyrase, anti-platelet protein, calreticulin, antigen 5 family proteins were identified and grouped on the basis of biological and functional roles. Secondly, differential protein expression and annotations between salivary glands of sugar fed vs blood fed mosquitoes was analyzed using 2-Delectrophoresis combined with MALDI-TOF mass spectrometry. The alterations in the differential expression of total 38 proteins was observed out of which 29 proteins like beclin-1, phosphorylating proteins, heme oxygenase 1, ferritin, apoptotic proteins, coagulation and immunity like, serine proteases, serpins, c-type lectin and protein in regulation of blood feeding behavior were found to be up regulated while 9 proteins related to blood feeding, juvenile hormone epoxide hydrolase ii, odorant binding proteins and energy metabolic enzymes were found to be down regulated. To our knowledge, this study provides a first time baseline proteomic dataset and functional annotations of *An*. *culicifacies* salivary gland proteins that may be involved during the blood feeding. Identification of differential salivary proteins between sugar fed and blood fed mosquitoes and their plausible role may provide insights into the physiological processes associated with feeding behavior and sporozoite transmission during the process of blood feeding.

## Introduction

Malaria, a vector borne parasitic disease is caused by protozoa in the genus *Plasmodium* and affects 198 million cases and leads to an estimated 584,000 deaths worldwide in 2013 [1]. Among different anopheline vectors, *Anopheles culicifacies* is the most abundant rural vector of malaria in South East Asian region including India, where it contributes for about 70% of malarial cases [2–3]. For malaria transmission, various *Plasmodium* species are injected into the human host typically via the bites of female *Anopheles* mosquito. In order to transmit malaria, at least two bites are required by the mosquito, one for acquiring the parasite infection and other for the transmission of malaria parasites to a new human host [4].

The salivary glands of female *Anopheles* mosquitoes are important because infective form of malaria parasite must invade mosquito salivary glands, before they are transmitted to the human host. The salivary glands are also the site for the maturation of sporozoites, where a set of sporozoite-vector interactions takes place and these interactions determines the competence of parasite and success of malaria transmission [5]. Salivary glands of blood sucking insects have evolved to complement their blood feeding behaviour and produces large array of biochemically active molecules, which deactivates host’s hemostatic response triggered by biting and helps in food ingestion and digestion [6]. Salivary proteins of haematophagous arthropods have immunomodulatory, anti-inflammatory and anti-coagulant properties for successful blood feeding. In addition, saliva proteins are antigenic and immunogenic which boosts the infectivity of parasite [7–9]. For the intake of blood meal, female mosquitoes inject the concoction of these salivary molecules into the vertebrate host and this complex saliva mixture act as a transmission fluid for the parasite [10].

Earlier transcriptomic studies have contributed a lot in understanding the salivary gland tissue complex in various mosquito species [11–12] however today, rapid and more sensitive technological advances in proteomics approaches enable to identify large catalogues of annotated functional proteins and their molecular and biological characterization. Proteomics has its advantage over genomic studies as it fills the void between genome sequence and the fate of its expression at cellular level. In recent years by utilising the high throughput technique of mass spectrometry many proteomic maps of important mosquito species salivary gland have been generated which provided new insights into the functional role of various salivary proteins in *An*. *stephensi* [13], *An*. *campestris*-like [14], *An*. *barbirostris* species A2 [15], *Aedes aegypti* [16], *An*. *funestus*[17],*An*. *gambiae* [10, 18], *Culex pipiens quinquefasciatus* [19]. Studies on the differentially expressed salivary gland proteins of susceptible and insecticide resistant mosquitoes of *An*. *stephensi* have also been carried out earlier in our lab using 2D electrophoresis and mass spectrometry [20]. *An*. *culicifacies*, a rural vector of malaria in South East Asian region including India as a complex of five isomorphic sibling species A, B, C, D, and E [21]. Since few transcriptomic data are available for *An*. *culicifacies* mosquitoes and unknown genome sequences till date, therefore, a baseline information and detailed description of proteomics data of *An*. *culicifacies* salivary glands will serve as a platform for all the advanced future studies during development and transmission of malaria parasite.

In the present study, using an in solution and 1-DE in gel trypsin digestion approach followed by high throughput LC/MS/MS analysis and data mining, a first comprehensive proteomic catalogue of *An*. *culicifacies* salivary gland functional proteins with their functions and biological process is provided. We also provide data on annotated proteins of *An*. *culicifacies* salivary glandsusing 2-DE coupled with MALDI-TOF TOF mass spectrometry using blood fed mosquitoes. Our results revealed significant compositional differences in salivary gland proteins expressed after blood meal as compared with sugar fed control mosquitoes. This baseline proteomic catalogue of sugar fed *An*. *culicifacies* salivary glands may be further explored to elucidate a role of novel expressed proteins in parasite blood feeding and malaria transmission. This information will serve as a platform for all the advanced future studies related to blood feeding behaviors, parasite maturation in *An*. *culicifacies* mosquitoes to achieve the goal of developing malaria blocking strategies and other aspects of malaria transmission.

## Methods

### Chemicals

All chemicals were of analytical grade and were purchased from Sigma, St. Louis, MO, USA. Proteomic grade trypsin and protease inhibitors set was purchased from Roche Diagnostics, Germany. All kits and protein markers were purchased from Banglore Genei, Bangalore, India and G-biosciences, St. Louis, MO, USA. All the solutions were prepared using Milli-Q purified water, Milliore Corp., Bedford, MA, USA.

### Mosquitoes

*An*. *culicifacies* mosquitoes were used in this study. This strain has been successfully reared through consecutive generations in our insectaries at National Institute of Malaria Research, New Delhi, INDIA. Female mosquitoes aged 2–3 days were maintained in stable conditions of temperature 27°C ± 2°C and 70%± 10% relative humidity and a photoperiod of 12:12 (light/dark) hours. These mosquitoes were divided into two groups i.e. sugar fed (SF) and blood fed (BF) mosquitoes. SF mosquitoes were fed on 10% sucrose diet only whereas the BF mosquitoes were fed on blood meal by feeding on rabbits. The protocol for mosquitoes feeding on rabbits was approved by the animal ethics committee of National Institute of Malaria Research, India.

### Salivary glands extract (SGE) preparation

Salivary glands were dissected from 100 adult cold anesthetized *An*. *culicifacies* female mosquitoes (SF and BF each) under stereomicroscope (4X magnification) using fine needles and pooled in Phosphate Buffered Saline (PBS; 10mM Na_2_SO_4,_ 145mM NaCl (pH 7.2) containing protease inhibitors (Complete, Roche Diagnostics, Germany). 100 pairs of pooled salivary glandswere homogenised by ultrasonication (3 pulses of 20 sec each) on ice. Salivary gland homogenate suspension was then centrifuged for 10 min at 5000 rpm at 4°C. Cell debris pellet was discarded and supernatant containing salivary gland protein extract (SGE) was stored at -80°C until further investigations. Protein concentration in the SGEs was quantified by Lowry’s method (GeNei^TM^ Protein Estimation Kit) using BSA as a standard [22].

### Sample preparation for LC-MS/MS

#### In solution trypsin digestion

SGEs were reduced, alkylated and digested with trypsin for LC-MS/MS analysis. Briefly, 50 μg of SGE was denatured using 4M urea. After that disulphide bonds of the proteins were reduced by incubating at 56^0^ C for 1 hour with dithiothreitol (DTT, 10mM). After reduction, proteins were alkylated with iodoacetamide (IAA, 25mM) in dark for 30 min at 25^0^ C. Subsequently, ammonium bicarbonate (NH4HCO3, 100mM pH 8.1) was added to the protein solution to reduce the urea concentration to 0.5M. Finally, the protein lysates were digested in to peptides by incubating with trypsin: substrate ratio of 1:50 at 37°C overnight. This trypsin digest obtained was dried in a speed vac till complete dryness. Samples were cleaned and desalted using C18 packed ziptip for further analysis by nano LC-MS/MS.

#### One-dimensional gel electrophoresis (1-DE)

1-DE experiments were performed for fractionation of SGE samples of SF mosquitoes. Briefly, 27 μg of SGE sample was mixed with sample buffer (0.625M TrisHCl, 10% SDS, glycerol, and distilled water) containing β-mercaptoethanol (10% vol/vol) and boiled for 5 min at 95°C. Denatured protein sample were then loaded on to SDS PAGE mini gel consisting of 3% stacking and 12% resolving gel of 1-mm thickness along with the protein molecular weight marker (Genei protein range marker, Bangalore Genei) and subjected to electrophoresis (Bio-Rad electrophoresis apparatus, USA). After completion of the run, the gel was stained with FOCUS-FAST silver^TM^ stain (G-Biosciences). Various bands were observed on the gel after silver staining. Each band was cut and collected in separate eppendorf tube in 50 μl of stop solution (2% acetic acid) and further stored at -20°C for trypsin digestion.

#### Two-dimensional gel electrophoresis (2-DE)

For 2-DE analysis, SGEs of both SF and BF mosquitoes were desalted and cleaned using the ReadyPrep 2D Cleanup Kit (Bio-Rad). 100μg of each sample was resuspended in 300μl of rehydration buffer (8M urea, 2M thiourea, 2% CHAPS, 50 mM DTT, 0.2% Bio-Lyte 3/10 ampholyte and a trace of bromophenol blue). Samples of both SF and BF mosquitoes were then immobilized on 17 cm IPG strips of pH 3–10 using a Protean IEF Cell (Bio-Rad) and proceeded for isoelectric focusing (IEF). Briefly, each sample was pipetted as a line along the edge of a rehydration tray channel. IPG strips were then gently placed on to the sample in rehydration tray (gel side down) and layered with 2–3 ml of mineral oil. The samples were rehydrated on IPG strips at 20°C overnight. After that IEF was run in three steps: 1) 250V for 20 min in linear mode; 2) 10000V for 2.5 hrs in linear mode; 3) 10000V for 5–7 hrs and 40000 V-hrs in rapid mode. After IEF run, the strips were equilibrated first in equilibration buffer I (6M urea, 0.375M Tris-HCl, pH 8.8, 2% SDS, 20% glycerol and 2% DTT) for 10 min and then in equilibration buffer II (6M urea, 0.375M Tris-HCl, pH 8.8, 2% SDS, 2 0% glycerol and 2.5%, IAA for 10 min. The second dimension separation was carried out on 10% SDS-PAGE on Mini Protean cell (Bio-Rad). The 2-DE gels were silver stained with FOCUS-FAST silverTM stain according to manufacturer’s instructions (G-Biosciences) after the run to visualize the spots and scanned using HP Scanjet G4010.

#### 2-DE gel analysis

ImageMaster 2D Platinum 7.0 software (GE Healthcare Life Sciences) was used for 2-DE gel comparative analysis. Gels of SF and BF samples were analyzed and all the differentially expressed spots in blood fed samples were excised and collected in separate eppendorf tubes in 50 μl of stop solution (2% acetic acid) and stored at -20°C for trypsin digestion and MALDI-TOF analysis.

#### In gel trypsin digestion

Silver stained gel pieces of all the bands and selected 2-DE spots were destained until the gel became translucent. Gel pieces were dehydrated using acetonitrile (ACN) and completely dried in a speed vac. These dried gel pieces were incubated with 10mM DTT in 100mM NH_4_HCO_3_ for 1 hour at 56^0^ C for reduction of proteins. For subsequent alkylation, DTT was removed and 55mM IAA in 100mM NH_4_HCO_3_ was added and the solution was kept for 45 min at ambient temperature in dark with occasional vortexing. Afterwards, the gel pieces were washed with 100mM NH_4_HCO_3_ (50μl) for 10 min and dehydrated with ACN two times and gel pieces were completely dried. Digestion buffer (50mM NH_4_HCO_3_ and 5mM CaCl_2_) was then added to gel pieces along with trypsin (12.5ng/μl) in an ice cold bath for 45 min. The gel pieces were extracted thrice with extraction buffer (5% formic acid in 50% acetonitrile, 20 min for each change) at room temperature. All the samples of SDS-PAGE gel bands were vacuum dried and reconstituted in 2% acetonitrile with 0.1% formic acid and all the 2D spots samples were suspended in Tris Acetate (TA) buffer for further LC/MS/MS and MALDI-TOF analysis.

### Mass spectrometry and protein identification

#### Nano LC-MS/MS

Mass spectrometric analysis of in-solution and 1-DE in-gel protein digests (bands) of SF samples was performed on Thermo Scientific™ LTQ XL™ ion trap mass spectrometer. Each sample (15μl) was injected onto a New Objective PicoFrit C18 nanospray column of 360 um OD x 75μm ID x 15μm tip opening dimensions using a Thermo Scientific Surveyor Auto sampler operated in the no waste injection mode. The flow rate was 300nl/min and a linear acetonitrile gradient was used to separate the tryptic peptides based on their hydrophobicity. For in-solution digests, 2 to 35% linear acetonitrile gradient (98–65% water) with 0.1% formic acid was used over 210 min and for in-gel digests 2 to 32% acetonitrile gradient (98–68% water) with 0.1% formic acid was used over 85 min followed by high and low organic washes for another 5 min via a nanospray source. All spectra were obtained in a positive ion mode with the spray voltage set to 1.8kV and the ion transfer capillary set at 180°C. A data-dependent top 5 method was used for peptide sequencing where a full MS scan from m/z 350–1700 was followed by MS/MS scans of the five most abundant ions. Each ion was subjected to CID (Collision Induced Dissociation) for fragmentation and peptide identification. MS/MS threshold was 500 counts. Raw m/z data files derived from LC-MS/MS were analyzed using Proteome Discoverer 1.4 (Thermo Scientific). SEQUEST algorithm was used against the most recent species-specific fasta database for mosquito from NCBInr/UniProt (http://www.uniprot.org). The input parameters used for the search were dynamic modification: oxidation of methionine, static modification: carbamidomethylation of cysteine, enzyme: trypsin, missed cleavages: 2, precursor mass tolerance: ± 5000 ppm, fragment mass tolerance: ± 2 Da and the peptide level filter were used for high confidence peptides only. Results were further interpreted manually and proteins were selected on the basis of score, sequence coverage and number of peptides.

#### MALDI TOF/TOF MS

Trypsin digested 2 DE spots samples were first desalted and concentrated on C18 Zip Tips (Millipore, USA). Desalted peptides samples were mixed with α-cyano-4-hydroxy cinnamic acid matrix in 1:1 ratio and the 2μl of this mixture was spotted on to the MALDI plate. The plate was analyzed on MALDI TOF/TOF Bruker Daltonics UltraFlex III instrument operated in positive-ion reflector mode of 500–3000m/z detection range. Further analysis was done with Flex Analysis^TM^ software (Bruker-Daltonics) and calibrated internally for autoproteolysis of peptides with trypsin to obtain the peptide mass fingerprint. Peaklist data files obtained were analyzed using peptide mass fingerprinting (MatrixScience) search against most recent mosquito database from UniProt for identification of the proteins. Parameters used for search were: fixed modification (carbamidomethyl), variable modification (Methionine oxidation), enzyme (trypsin), peptide tolerance: 100-500ppm, Missed Cleavages: 1 or 2.

### Bioinformatics and Data analysis

Bioinformatics analysis were carried out using Blast P and SMART algorithm (http://smart.embl-heidelberg.de/) in order to find out functions and conserved domains. Signal peptides were also depicted using Signal P 4.1 (http://www.cbs.dtu.dk/services/SignalP/). Gene ontology (GO) search engines (http://www.geneontology.org/) were used for further identification of biological process and molecular function of all identified annotated putative functional proteins. The cellular component was predicted using CELLO (http://cello.life.nctu.edu.tw/)[23] and GO software.

The mass spectrometry proteomics data have been deposited to the ProteomeXchange Consortium [24] via the PRIDE partner repository with the dataset identifier PXD003450.

## Results

**Fig 1:**
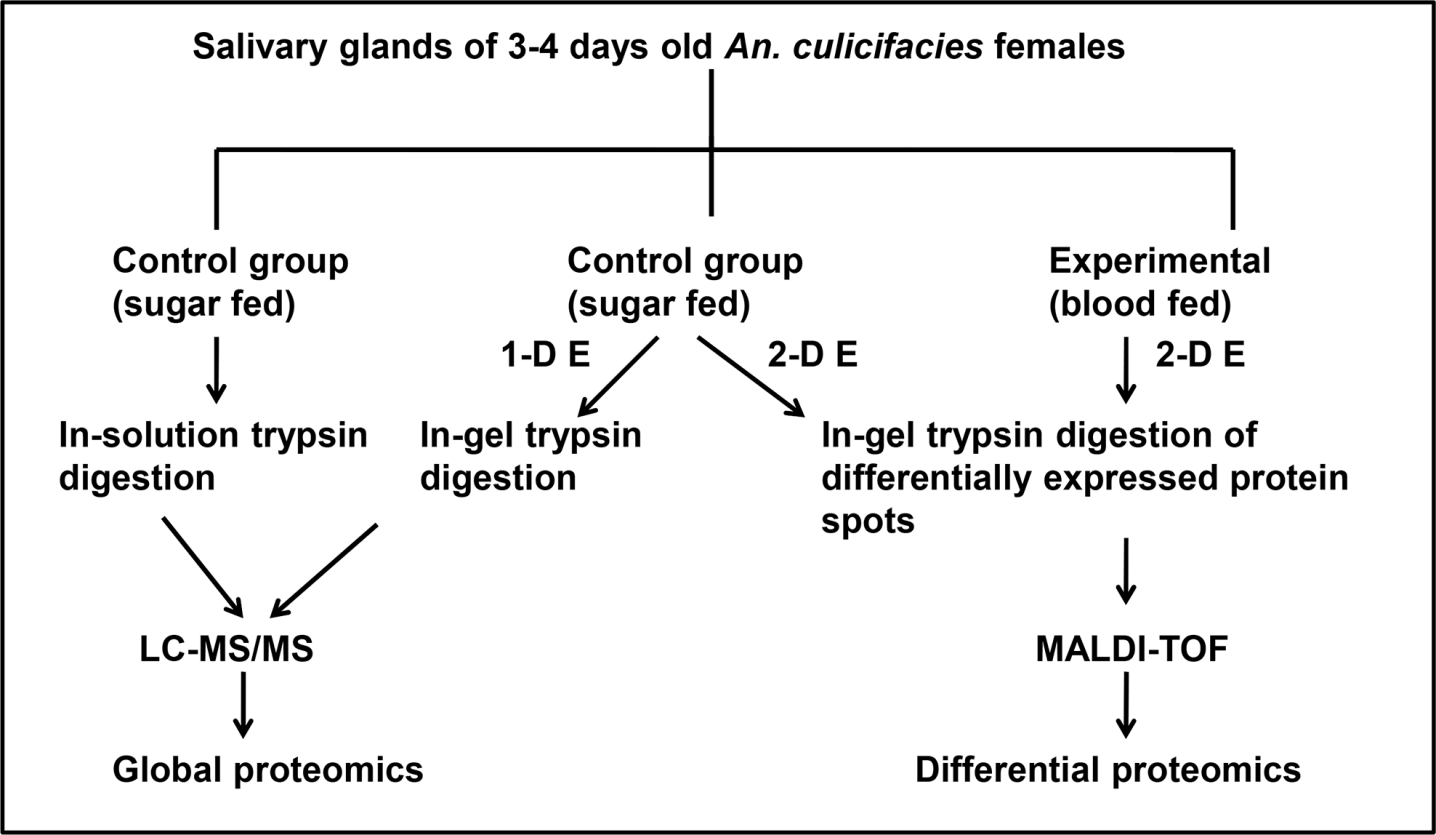
Experimental design. A schematic depiction of global and differential proteomic experiments of salivary gland of sugar fed (SF) and blood fed (BF) *An*. *culicifacies* mosquitoes.

In order to understand the importance and functional role of novel expressed or annotated mosquito salivary gland proteins following blood meal, we have carried out our analyses on two aspects (Fig 1). Firstly, since no proteomic data is available for *An*. *culicifacies*, we aimed to prepare the baseline proteomic repertoire of salivary gland proteins of female *An*. *culicifacies* mosquitoes. In this study, a proteomic approach, 1-DE coupled with nano LC-MS/MS, was used for identification of salivary gland proteins of *An*. *culicifacies*. Secondly, differential proteomic analysis using 2-DE coupled with MALDI-TOF mass spectrometry was carried outto implicate the functional roles of novel expressed and annotated proteins following blood meal.

### Characterization of salivary gland proteome of female *An*. *culicifacies*: in- solution and in-gel approach

**Fig 2:**
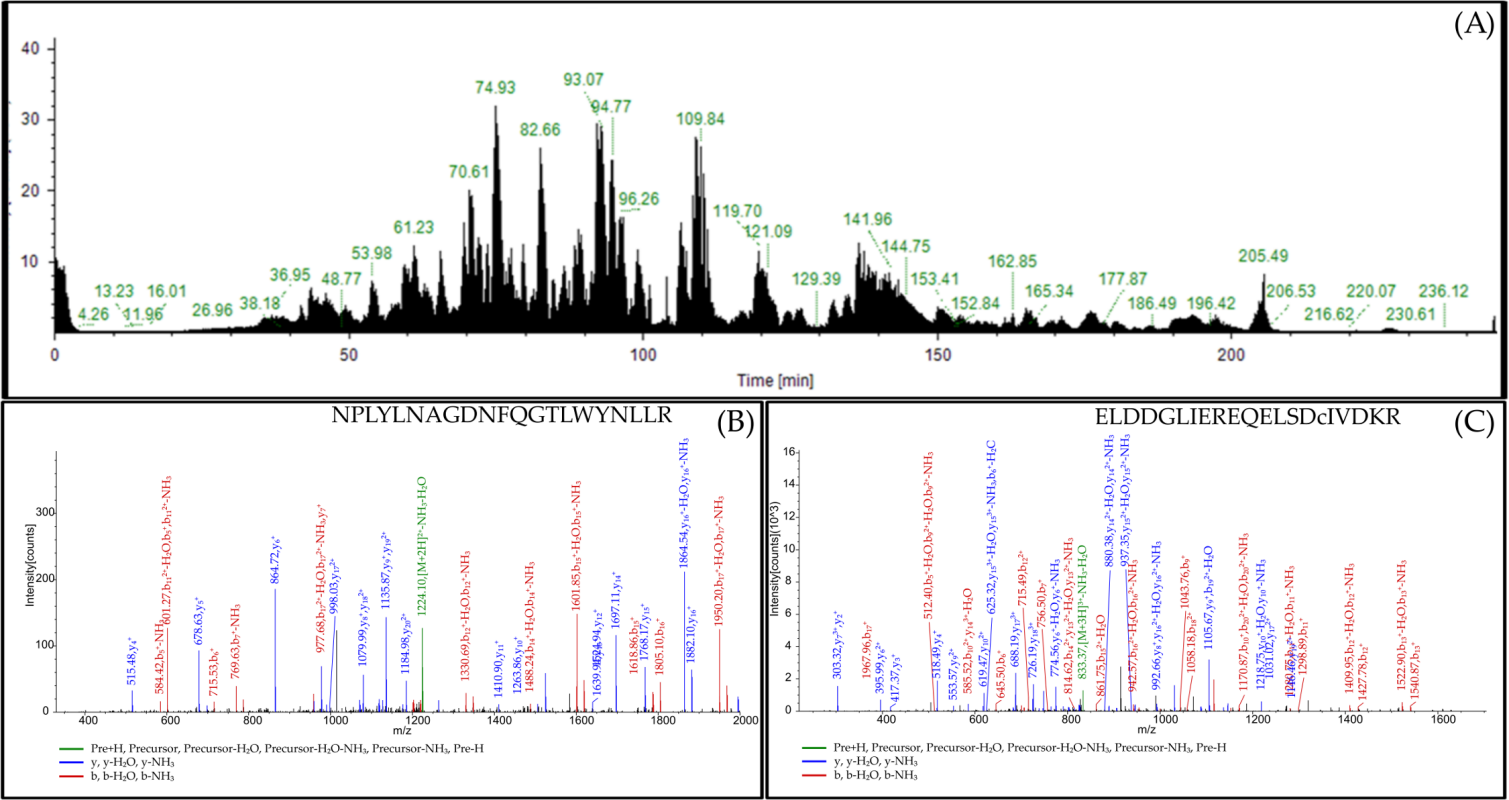
Characterization of salivary gland proteome of *An*. *culicifacies* using in-solution approach. **(A)** Total Ion chromatogram (LC/MS/MS) of in solution trypsin digest salivary gland proteins **(B)** Product ion MS/MS spectrum with peak at m/z1242.46 corresponds to the peptide sequence NPLYLNAGDNFQGTLWYNLLR which matched to known protein salivary apyrase of *An*. *stephensi*. **(C)** MS/MS spectrum of a peak at m/z845.46 corresponds to peptide ELDDGLIEREQELSDcIVDKR matched with antiplatelet protein of *An*. *gambiae*.

The aim of this study was to identify and characterize total salivary gland proteins of *An*. *culicifacies* to provide a baseline proteomic database as an initial step towards the cataloging of proteins and peptides. Analysis of salivary gland proteome of *An*. *culicifacies* using in-solution approaches leads to the identification of 81 major proteins. A total ion chromatogram (TIC) of the run obtained of in–solution digested sample is shown (Fig 2A). A representative MS/MS spectrum of two anti-hemostatic salivary gland specific proteins i.e. salivary apyrase protein peptide NPLYLNAGDNFQGTLWYNLLR with peak at m/*z* 1242.46 and antiplatelet protein peptide ELDDGLIEREQELSDcIVDKR with peak at m/z845.46 are depicted in Fig 2B and 2C.

**Fig 3:**
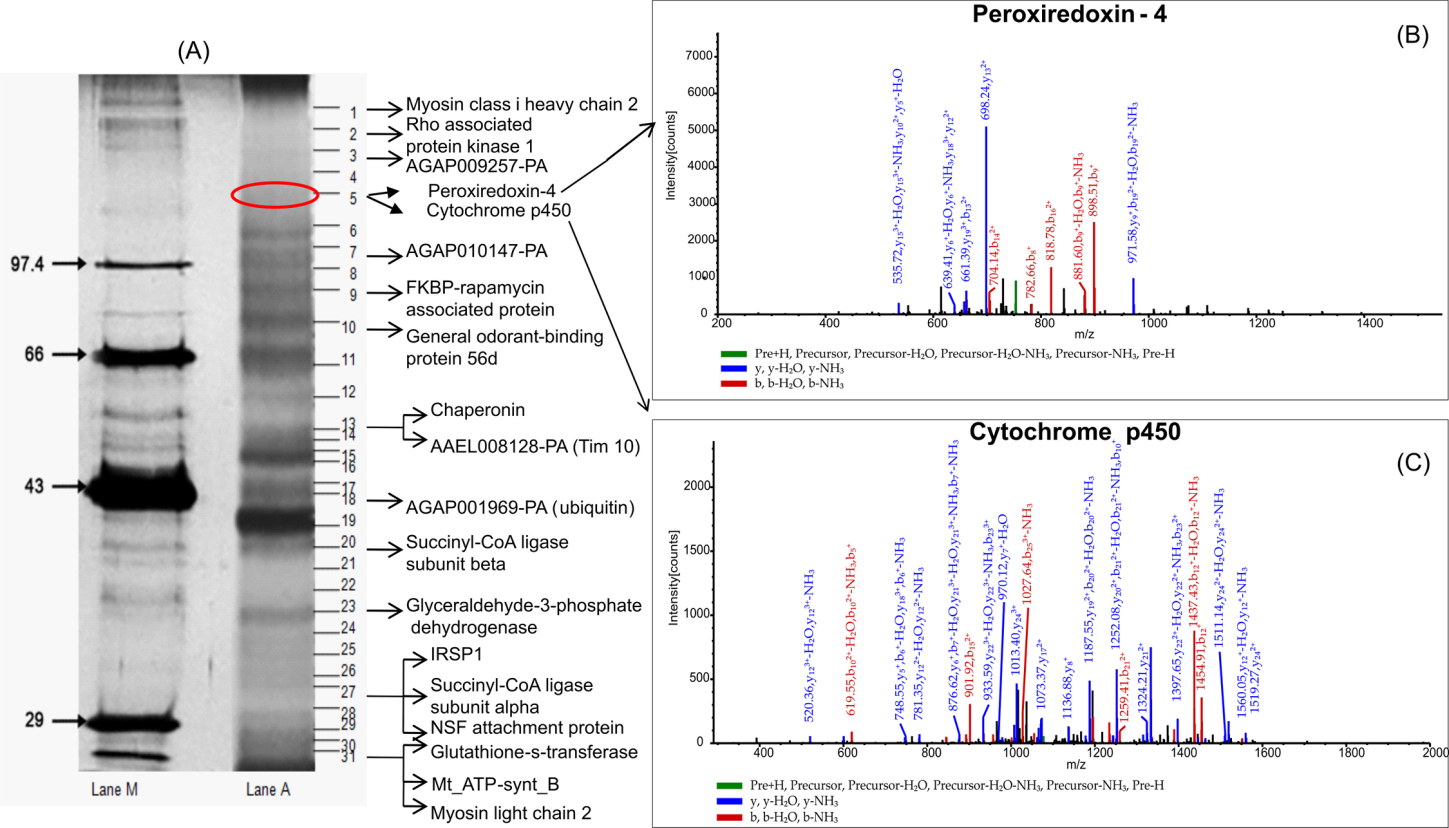
Characterization of salivary gland proteome of *An*. *culicifacies* using 1-DE gel based approach. **(A)** 1-DE gel profile of salivary gland proteins after silver staining. Lane A: Salivary glands extract. Lane M: marker (size 14 to 100 kD) (B) MS/MS spectrum of peptide DNPEYFQKVMNSPHLLSKmDQYNFFR with peak at m/z1089.99 corresponds to protein AGAP002197-PA (cytP450) which is orthologous protein of *An*. *gambiae*. (C) MS/MS spectrum of peptide KEGGLGKINIPLVSDITHSIAK with peak at m/z765.33 corresponds to protein orthologous to a Peroxiredoxin-4 of *Culex tarsalis*.

1-DE analysis of *An*. *culicifacies* salivary gland protein extract by in-gel approach showed 31 major well resolved bands on the gel after silver staining (Fig 3A). To identify the proteins in each band, each individual band was excised, digested with trypsin and subjected to nano LC/MS/MS analysis and subsequent analyses using SEQUEST algorithm leads to the identification of 36 proteins. Of the 36 proteins, 11 proteins were found to be common as identified by in solution approach. Important proteins identified by SEQUEST algorithm corresponding to each band are shown in Fig 3A. Representative MS/MS spectrum of proteins identified under band 5 with peak m/z i.e. AGAP002197-PA (cytP450) and Peroxiredoxin-4 were shown respectively (Fig 3B and 3C).

**Fig 4:**
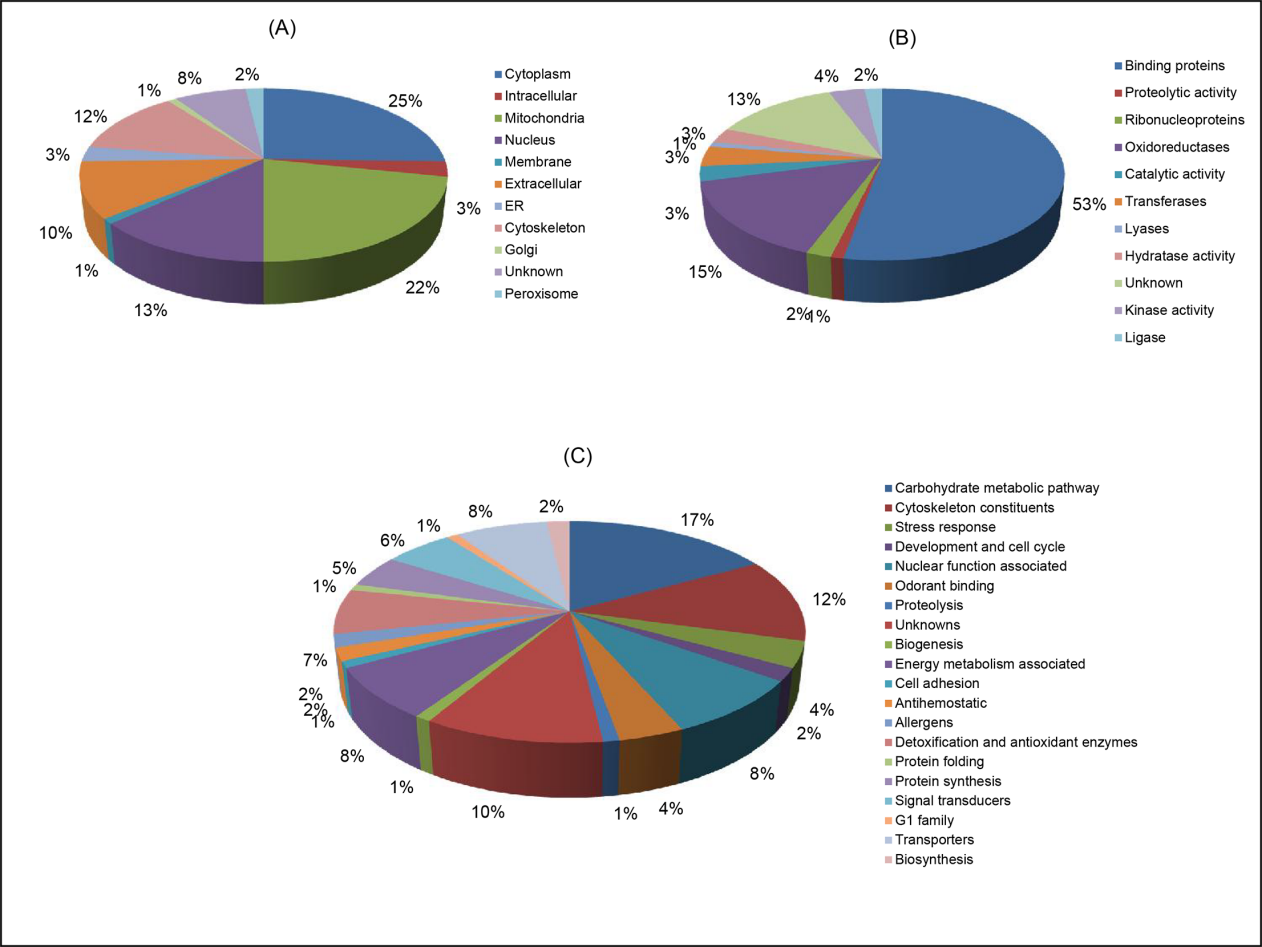
Functional annotations of *An*. *culicifacies* salivary gland proteome using Gene Ontology tools. (A) Sub cellular location (B) Molecular function (C) Biological process

Hence, a total 106 putative functional proteins were identified using both in solution and in gel digestion approach in *An*. *culicifacies* salivary glands. Importantly, salivary gland specific proteins like D7r1, D7r2, D7r4, salivary apyrase, anti-platelet protein, antigen 5 family proteins, G1 family long form salivary protein 3, salivary maltase and other proteins like calreticulin, prohibitin, catalase, peroxiredoxin-4, cytochrome P450, thioredoxin, Glutathione S transferase were identified. Molecular functions, biological processes and sub cellular localization of proteins were further studied using various bioinformatics algorithms and gene ontology assignments. Most of the proteins were found to be present at cytoplasm (25%) followed by mitochondria (22%), nucleus (13%), extracellular (10%), cytoskeleton (12%), unknown (8%), etc. (Fig 4A). On the basis of molecular function almost 53% proteins were scrutinized to be the binding proteins followed by oxidoreductases (15%), unknowns, transferases, hydratases, kinases, ligases, catalytic enzymes, ribonucleoproteins and proteolytic enzymes (Fig 4B). Functional putative proteins were classified into 20 groups according to the biological role and among them mostly proteins were assigned to the carbohydrate metabolic pathway (17%) followed by cytoskeleton constituents (12%), unknowns (10%) etc. (Fig 4C). All the identified 106 proteins were also subjected to domain based functional analysis and are summarized below in Table 1.

**Table 1:** Salivary gland protein profile of sugar fed *An*. *culicifacies* using in-solution and in-gel approach.

Accession	Protein	In- solution	In-gel	Organism	% Sequence coverage	Peptide matches	Comment / Band No. (in-gel)
**Cytoskeletal: 13**							
T1DPM1	Putative actin indirect flight muscle	✓		*An*. *aquasalis*	6.22	17	Actin
Q7PJV2	AGAP010147-PA	✓	✓	*An*. *gambiae*	40.68	82	Myosin head / Band 14
Q7PSI4	AGAP010929-PA	✓		*An*. *gambiae*	52.80	15	Tubulin
F5HME9	AGAP001797-PE	✓	✓	*An*. *gambiae*	46.32	13	Tropomyosin/ Band 13
F5HMN8	AGAP001497-PB	✓		*An*. *gambiae*	19.73	13	Calponin homology domain
B0WAN1	Paramyosin, long form	✓		*Cu*. *quinquefasciatus*	16.78	12	Myosin tail
Q7PNL5	AGAP005459-PA	✓		*An*. *gambiae*	30.15	3	Chitin
B0WVV0	Putative uncharacterized protein	✓		*Cu*. *quinquefasciatus*	4.47	2	Spc97_Spc98 domain
T1E7U1	Putative troponin t skeletal muscle	✓	✓	*An*. *aquasalis*	14.44	5	Troponin/ Band 18
B3RH54	Lava lamp protein	✓		*Cu*. *quinquefasciatus*	2	3	Microtubule associated
Q7Q7K5	AGAP011515-PA		✓	*An*. *gambiae*	24.43	6	Actin/ Band 18
E9P4K4	Myosin light chain 2		✓	*Cu*. *pipiens*	26.67	5	EF-Hand domain/ Band 31
Q7Q978	AGAP004877-PA		✓	*An*. *gambiae*	14.17	3	Myosin
**Energy metabolism related: 08**							
Q17FL3	ATP synthase subunit beta	✓	✓	*Aedes aegypti*	65.48	21	ATP binding/ Band 18
Q7PHI8	ATP synthase subunit alpha	✓	✓	*An*. *gambiae*	27.22	11	ATP binding / Band 17
Q9XYC8	AAEL005798-PA	✓		*Aedes aegypti*	29.44	8	V ATPase
A7UTS9	AGAP005627-PC	✓		*An*. *gambiae*	23.10	6	ATP guanido P Transferase
Q7PWZ7	AGAP001138-PA	✓	✓	*An*. *gambiae*	12.71	2	Mt. ATP synthase/ Band 31
Q7PNG6	AGAP007841-PA	✓		*An*. *gambiae*	28.40	3	ATP synthase epsilon
Q7Q3N8	ATP synthase subunit gamma	✓	✓	*An*. *gambiae*	14.14	3	ATP synthase/ Band 27
Q7Q5G0	AGAP006456-PA		✓	*An*. *gambiae*	15.65	3	NADH dehydrogenase (DH)/ Band 29
**Carbohydrate metabolism: 18**							
Q7Q1U8	Glyceraldehyde-3-phosphate DH	✓	✓	*An*. *gambiae*	31.63	6	Phosphorylation/ Band 23
Q7Q3F6	AGAP007852-PA	✓		*An*. *gambiae*	24.30	13	Aconitase
Q7PSR9	AGAP011159-PA	✓		*An*. *gambiae*	50.98	5	Cytochrome C
Q7Q3L6	Phosphorylase	✓		*An*. *gambiae*	8.31	4	Phosphorylation
Q7PYE7	AGAP001903-PA	✓	✓	*An*. *gambiae*	50.74	9	L_DH/ Band 27
Q7Q3D8	AGAP007827-PA	✓		*An*. *gambiae*	22.40	4	Enolase
T1DQ50	Putative isocitrate DH	✓		*An*. *aquasalis*	15.36	3	Oxidoreductase
T1E9K5	Putative 2-oxoglutarate DH e1	✓		*An*. *aquasalis*	7.14	5	Oxidoreductase
F5HKV6	Fructose-bisphosphate aldolase	✓		*An*. *gambiae*	13.77	3	Aldolase
Q7PPE7	Pyruvate kinase	✓		*An*. *gambiae*	16.99	4	Phosphorylation
Q7PYD5	AGAP001884-PA	✓		*An*. *gambiae*	14.91	4	Fumarase C
B0VZW3	Glycerol-3-phosphate DH	✓		*Cu*. *quinquefasciatus*	18.36	4	Dehydrogenase
Q06DJ2	Salivary maltase	✓		*An*. *funestus*	19.90	3	Glycoside hydrolase family 13
Q7PV48	Citrate synthase	✓		*An*. *gambiae*	18.45	6	Transferase
Q7PQM3	6-phosphogluconate DH	✓		*An*. *gambiae*	14.73	5	Oxidoreductase
A7URV6	AGAP006936-PB	✓		*An*. *gambiae*	30.64	4	Cytochrome C1
B0XGN1	Succinyl-CoA ligase subunit alpha		✓	*Cu*. *quinquefasciatus*	10.53	2	Ligase/ Band 27
Q7PMT2	Succinyl-CoA ligase subunit beta		✓	*An*. *gambiae*	9.64	2	Ligase/ Band 20
**Transport: 08**							
Q7PPA5-2	Isoform A of Calcium-transporting ATPase	✓		*An*. *gambiae*	32.87	22	Cation Atpase
Q27238	ADP, ATP carrier protein 1	✓		*An*. *gambiae*	39.20	10	Mt carrier protein
Q7PMF3	AGAP010025-PA	✓		*An*. *gambiae*	7.45	2	Rab GDP dissociation inhibitor
F5HK77	AGAP002354-PB	✓		*An*. *gambiae*	5.67	2	Membrane trafficking
T1EB45	Putative endocytosis/signaling protein ehd1	✓		*An*. *aquasalis*	9.11	4	Dynamin domain
Q7PW34	AGAP009105-PA	✓		*An*. *gambiae*	4.77	2	HEAT repeats
Q1HRD0	AAEL008128-PA		✓	*Aedes aegypti*	21.35	1	Tim10 domain Band 13
B0X105	Soluble NSF attachment protein		✓	*Cu*. *quinquefasciatus*	8.53	2	NSF attachment protein/ Band 27
**Biosynthesis: 02**							
Q7QA89	AGAP004366-PA	✓		*An*. *gambiae*	15.88	6	Aldehyde DH
Q7Q3R0	AGAP007990-PA	✓		*An*. *gambiae*	6.51	2	UDP-glucuronosyl transferase
**Protein synthesis: 05**							
T1DN37	Elongation factor 1alpha	✓	✓	*An*. *aquasalis*	30.48	5	Band 18
P33514	40S ribosomal protein	✓		*An*. *gambiae*	27.60	2	Ribosomal_S7e
Q7PNJ7	AGAP000883-PA	✓		*An*. *gambiae*	10.54	3	Elongation Factor
T1DJH7	Putative elongation factor 2	✓		*An*. *aquasalis*	8.54	5	GTP binding
T1DFZ6	60s acidic ribosomal protein		✓	*An*. *aquasalis*	15.96	2	Ribosomal L10/ Band 25
**Nuclear function Associated: 09**							
Q7Q2C5	Histone H2B	✓		*An*. *gambiae*	37.36	3	Nucleosome assembly
B6DE21	Histone H3	✓		*An*. *darling*	30.15	2	Nucleosome assembly
Q7QAJ4	AGAP003671-PA	✓		*An*. *gambiae*	9.28	2	Homeobox domain
F5HM53	AGAP004028-PB	✓		*An*. *gambiae*	2.28	2	ResIII domain
B8RJF1	Histone H4	✓		*Culex tarsalis*	50.52	5	Nucleosome assembly
Q7QE14	AGAP010700-PA	✓		*An*. *gambiae*	4.68	3	HAND domain
Q7QAK7	DNA-directed RNA polymerase	✓		*An*. *gambiae*	3.83	3	Beta subunit
B0X8G8	RNA-binding motif protein		✓	*Cu*. *quinquefasciatus*	13.87	2	Band 29
B0XH66	Guanine nucleotide binding		✓	*Cu*. *quinquefasciatus*	10.80	4	NCD*/ Band 31
**Development and cell cycle related related: 02**							
Q7PMG2	AGAP009642-PA	✓		*An*. *gambiae*	7.74	2	Prohibitin
B0WN45	Putative uncharacterized protein	✓		*Cu*. *quinquefasciatus*	10.65	2	Borealin domain
**Detoxification and antioxidant enzymes: 07**							
Q8MUR9	Glutathione S-transferase S1-2	✓		*An*. *gambiae*	48.21	5	Transferase/ Band 31
Q5TX96	AGAP002197-PA		✓	*An*. *gambiae*	7.07	2	Cyt P450/ Band 5
B0XGK0	Glutathione S-transferase, theta		✓	*Cu*. *quinquefasciatus*	10.96	2	Transferase/ Band 31
T1E7E5	Dihydrolipoyl dehydrogenase	✓		*An*. *aquasalis*	15.22	3	oxidoreductase
B0XCN4	L (2) long form	✓		*Cu*. *quinquefasciatus*	8.96	5	Thioredoxin/ **Signal P: 1–29**
Q6RBZ5	Catalase	✓		*An*. *gambiae*		2	Peroxidase
B8RJA9	Peroxiredoxin-4		✓	*Cu*. *tarsalis*	16.30%	2	Peroxidase/ Band 5
**Stress response: 04**							
Q7PQK5	AGAP004192-PA	✓		*An*. *gambiae*	13.81	6	HSP 70/ **Signal P: 1–20**
Q7PT10	Heat shock protein 83	✓		*An*. *gambiae*	12.36	6	HSP 90
B0X2F8	FKBP-rapamycin associated protein		✓	*Cu*. *quinquefasciatus*	18.95	1	Kinase/ Band 9
B0WWW	Chaperonin		✓	*Cu*. *quinquefasciatus*	11.73	1	HSP 60/ Band 13
**Signal Transduction: 06**							
B0WM74	Putative uncharacterized protein	✓		*Cu*. *quinquefasciatus*	3.99	2	Pleckstrin homology domain
A0NBC2	AGAP007643-PA	✓		*An*. *gambiae*	17.34	3	14-3-3 family
Q16ZM1	AAEL008141-PA	✓		*Aedes aegypti*	3.38	3	PAS domain
Q17HK0	AAEL002654-PA	✓		*Aedes aegypti*	4.33	2	Growth factor receptor/ **Signal P: 1–24**
Q16J24	AAEL013466-PA	✓		*Aedes aegypti*	2.82	2	Ankyrin repeats
Q7PUN2	AGAP001969-PA		✓	*An*. *gambiae*	32.36	2	Ubiquitin domain/ Band 18
**Anti-hemostatic proteins:02**							
Q8I6Q2	Salivary apyrase	✓		*An*. *stephensi*	7.48	4	5'-Nucleotidase**/ Signal P: 1–22**
B3VDI9	Anti-platelet protein	✓		*An*. *gambiae*	8.97	4	Collagen binding
**Chemosensory/Odorant binding: 04**							
Q06DJ4	Short form D7r4	✓		*An*. *funestus*	17.58	3	**Signal P: 1–22**
O97414	D7r1 protein	✓		An. gambiae	6.31	2	**Signal P: 1–22**
Q95V98	Short form D7r2 salivary protein	✓		*An*. *arabiensis*	27.98	3	GOBP
B0X0G3	General odorant-binding protein 56d		✓	*Cu*. *quinquefasciatus*	9.16	2	GOBP/ Band 10
**SG1 family: 01**							
Q06DI5	G1 family long form salivary protein 3	✓		*An*. *funestus*	13.01	4	NCD*
**Allergens: 02**							
Q8I6R0	Salivary antigen-5 related protein	✓		*An*. *stephensi*	11.58	2	CAP domain**/ Signal P: 1–21**
L7RJB8	IRSP1		✓	*An*. *gambiae*	13.48	1	CAP domain/ Band 27
**Protein folding: 01**							
J7EQD2	Calreticulin	✓		*An*. *stephensi*	35.47	8	Multiple functions**/ Signal P: 1–16**
**Cell adhesion: 01**							
Q17HV9	AAEL002565-PA	✓		*Aedes aegypti*	2%	5	fibronectin 3
**Proteolysis: 01**							
Q5TP08	AGAP009917-PA	✓		*An*. *gambiae*	2.74	5	Rhs repeat
**Biogenesis: 01**							
Q17I93	AAEL002433-PA		✓	*Aedes aegypti*	13.92	2	Peroxisomal biogenesis factor 11/ Band 27
**Unknown role: 11**							
Q7PUV3	AGAP001622-PA	✓	✓	*An*. *gambiae*	27.83	5	EF- hand domain/ Band 31
Q5TR40	AGAP006179-PC	✓		*An*. *gambiae*	43.79	7	EF-hand domain
Q7Q515	AGAP006686-PA	✓		*An*. *gambiae*	6.16	9	EF-hand domain
F5HJ34	AGAP001023-PA	✓		*An*. *gambiae*	40.65	4	NCD*
Q174U1	AAEL006790-PA	✓		*Aedes aegypti*	3.90	3	NCD*
A7URJ0	AGAP007249-PB	✓		*An*. *gambiae*	19.85	2	NCD*
Q16FT3	AAEL014638-PA		✓	*Aedes aegypti*	16.24	2	NCD*/ Band 27
B0X0C8	Putative uncharacterized protein		✓	*Cu*. *quinquefasciatus*	13.51	2	NCD*/ Band 31
Q7PS70	AGAP003775-PA		✓	*An*. *gambiae*	10.82	2	NCD*/ Band 27
T1EB87	Uncharacterized protein		✓	*An*. *aquasalis*	10.46	2	NCD*/ Band 13
Q17N70	AAEL000785-PA		✓	*Aedes aegypti*	4.69	1	DUF1168/ Band 2

*NCD- No conserved domain

### Comparison of salivary gland proteins between Sugar Fed (SF) and Blood Fed (BF) *An*. *culicifacies* mosquitoes by 2-DE

**Fig 5:**
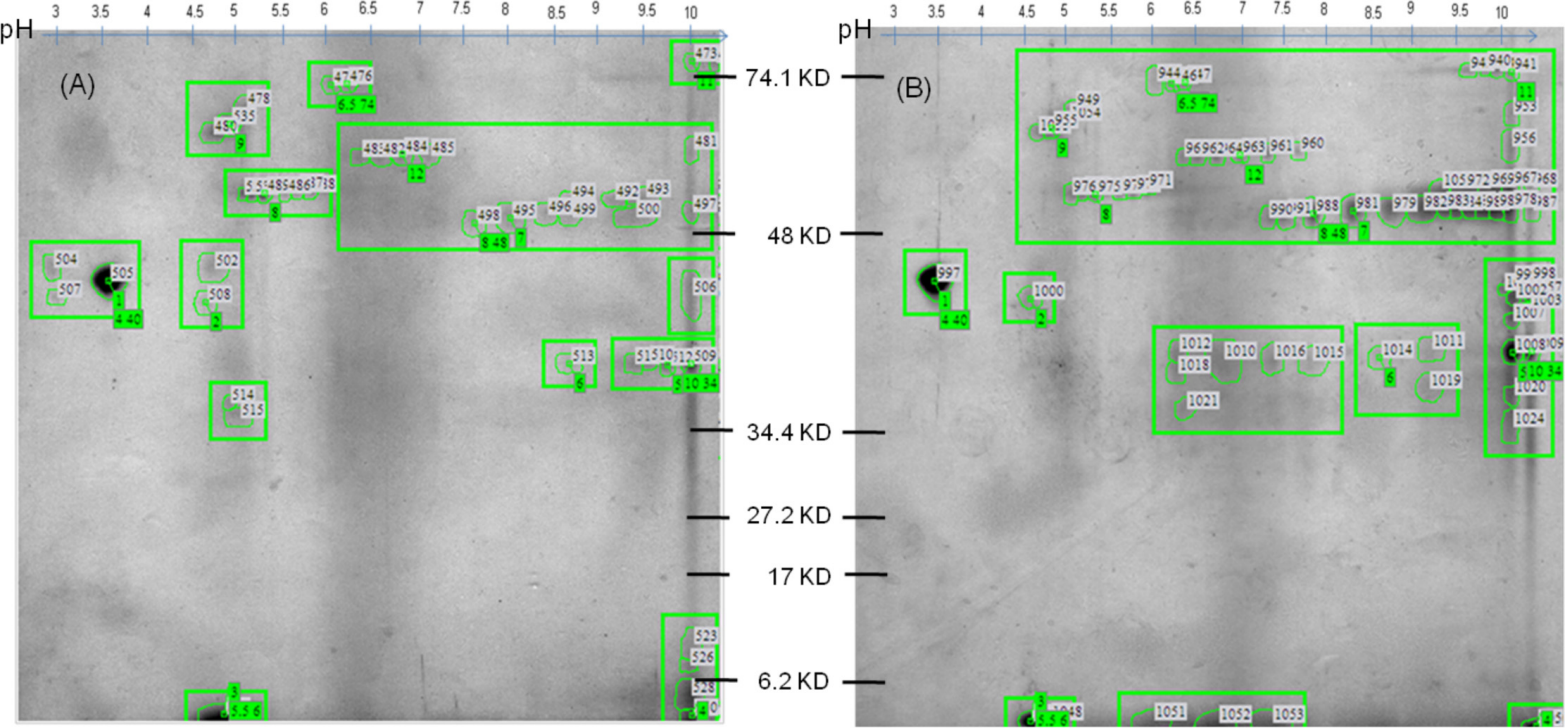
2-DE gel picture of *An*. *culicifacies* salivary gland proteins. **(A)** Gel picture of sugar fed species show total spot id number (473–535,black colored) **(B)** Gel picture of blood fed species show total spot id number (940–1058,black colored)

**Fig 6:**
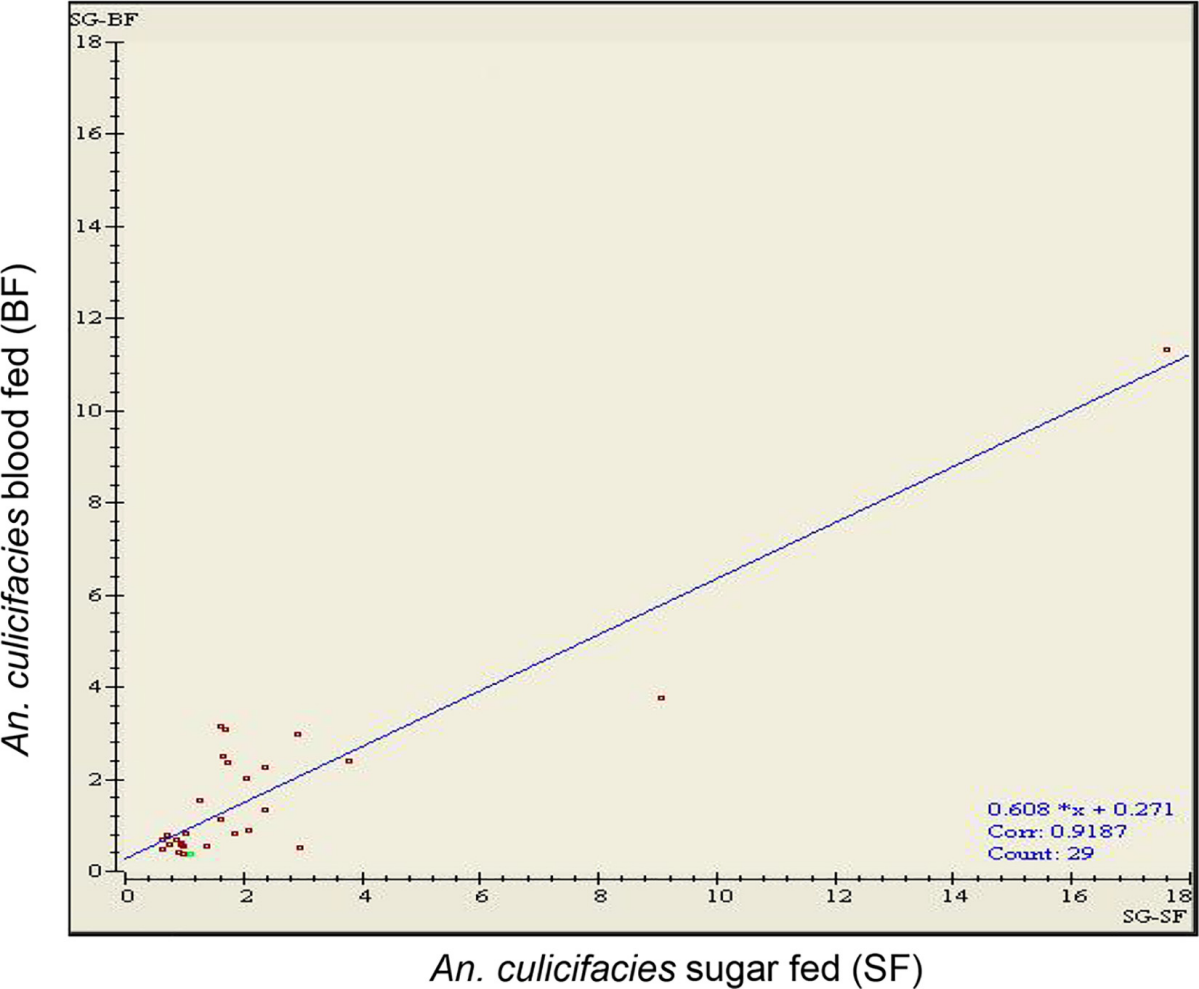
Scatter plot showing differential spots between SF and BF of *An*. *culicifacies*. Blue line shows linear regression. X axis: volumes of protein spots in SF species. Y axis: volumes of protein spots in BF species. Correlation coefficient was calculated and indicated at the bottom.

In order to identify the plausible implications of blood meal on the expression and annotations of salivary gland proteins, a comparative study between SF and BF mosquitoes using 2-DE coupled with MALDI-TOF TOF mass spectrometry was conducted in *An*. *culicifacies*. A total of 45 spots in SF and 71 spots in BF between a pI range of 4–10 and MW 6–80 kDa were detected using ImageMaster 2D Platinum software (Fig 5A and 5B). Among these total spots, 29 spots were found to be differential expressed between SF and BF mosquitoes. These differential spots in SF and BF salivary gland profile were compared on the basis of pixel volume of each spot. Pixel volume of spots of both SF and BF *An*. *culicifacies* are calculated on the basis of spot area and intensity and shown as a scatter plot with a correlation coefficient of > 0.91 between SF and BF (Fig 6).

**Fig 7:**
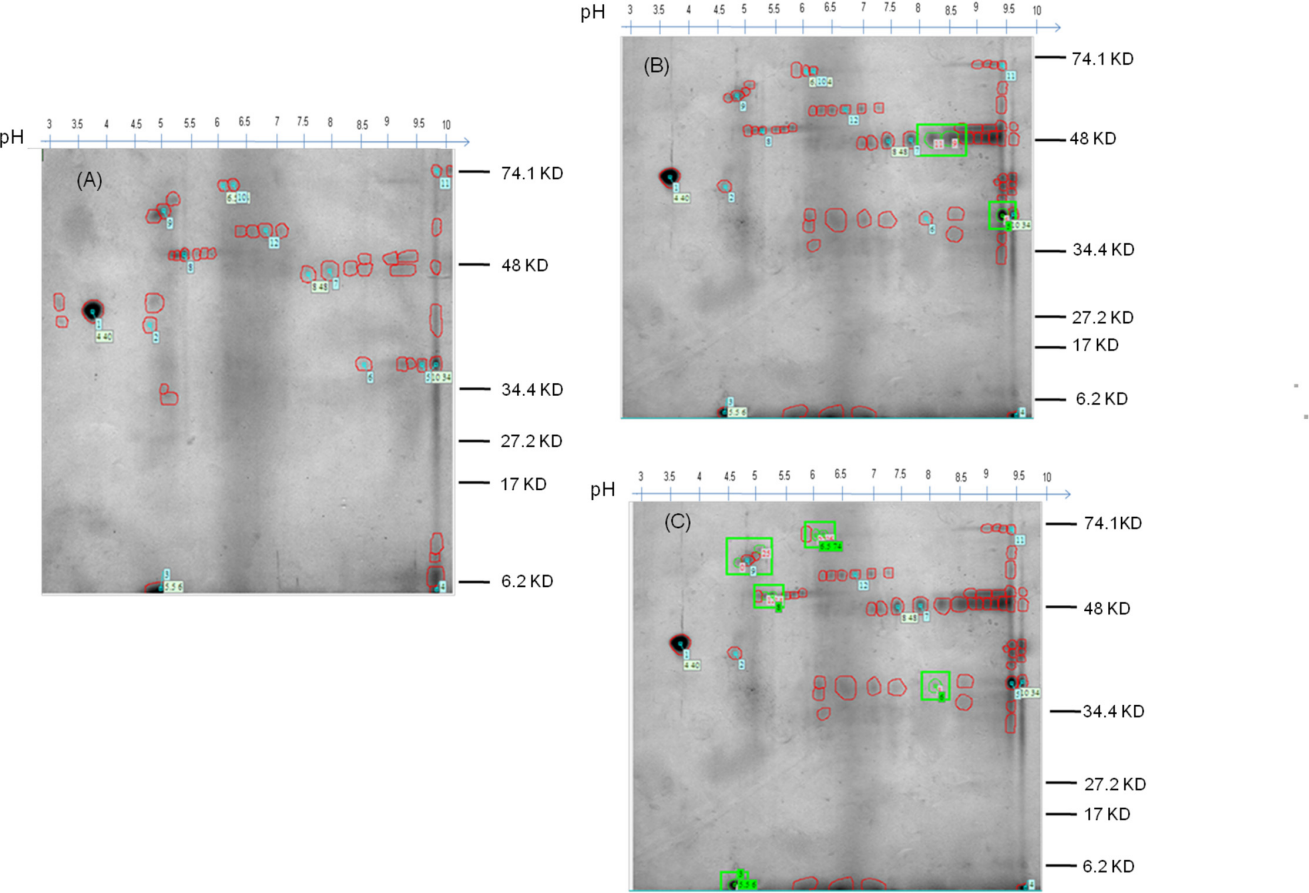
2-DE gel picture of annotated salivary gland proteins of BF *An*. *culicifacies*. (A) 2-DE salivary gland protein profile of sugar fed (SF) mosquitoes. (B) 2-DE salivary gland protein profile of over expressed spots (5, 9 11) in BF mosquitoes (green squared and numbered in red). (C) 2-DE salivary gland protein profile of under expressed spots (0, 2, 3, 12, 14, 25, 26, 27) in BF mosquitoes (green squared and numbered in red).

Among 29 annotated spots, only 11 spots were further processed for MALDI as remaining spots did not match the criteria for MALDI-TOF TOF mass spectrometry. Of the 11 differentially expressed spots 3 spots were found to be over expressed (match ID 5, 9, 11) and 8 spots were found to be under expressed (match ID 0, 2, 3, 12, 14, 25, 26, 27) in BF as compared to SF (Fig 7A–7C). Analysis of raw data files of all the 11 annotated spots using peptide mass fingerprinting (PMF) leads to identification of total 29 up regulated proteins and 9 down regulated proteins and their description with fold change are given in Table 2 and Table 3 respectively. All these proteins were further analysed using Gene ontology and other bioinformatics algorithms for the identification of molecular function.

**Table 2:** Annotated up regulated salivary proteins in *An*. *culicifacies* upon blood feeding.

Match ID/ Fold increase	Accession	Protein	Organism, % Sequence coverage	Peptide matches	Function/ Signal peptide if any
**5 / 1.94**	A0A023EV62	Putative ceramide kinase	*Ae*. *albopictus*, 15%	13	Phosphorylation
	A0A023EU04	Serine/threonine kinase	*Ae*. *albopictus*, 15%	9	Phosphorylation
	B0XFC4	Phosphatidylinositol transfer protein SEC14	*Culex quinquefasciatus*,14%	6	Signal transduction
	B0WIF0	Serine protease inhibitor	*Culex quinquefasciatus*,15%	9	Proteolytic
	C4N137	30 kDa salivary antigen family	*An*. *darling*, 16%	6	Antigen / Signal P: 1–29
	A0A023EDZ3	Putative secreted protein	*Ae*. *albopictus*, 35%	7	Signal P: 1–26
**9/ 1.79**	A0A084W2T3	Ribokinase	*An*. *sinensis*, 61%	8	Phosphorylation
	W5JB49	Calcium/calmodulin-dependent protein kinase 1	*An*. *darlingi* 30%	15	Phosphorylation
	A0A023EV08	Putative creatine kinase	*Ae*. *albopictus*, 28%	12	Phosphorylation
	B0X4Y5	Polo kinase	*Culex quinquefasciatus*, 19%	26	Phosphorylation
	A0A023EIY7	Tyrosine phosphatase iva1	*Ae*. *albopictus*, 32%	6	Phosphatase
	A0A084W4J8	MRAS2, putative	*An*. *sinensis*, 37%	14	Signal transduction
	Q7QGU9	Oxysterol-binding protein	*An*. *gambiae*, 23%	11	Signal transduction
	Q0IEK7	Lipoyltransferase 2, Mt	*Ae*. *aegypti*, 32%	11	Protein modification
	B0X918	Ferritin subunit	*Culex quinquefasciatus*, 37%	7	Iron homeostasis
	D3KAG7	CLIPB14	*An*. *gambiae*, 32%	9	Proteolytic
	A0A084VN17	AGAP004754-PA	*An*. *sinensis*, 63%	10	Caspase like domain
	W5J7X6	Glucose dehydrogenase	*An*. *darling*, 24%	9	Oxidoreductase
	J9E8U1	AAEL017136-PA	*Ae*. *aegypti*, 32%	13	Cyt P450
	A0A084WNZ9	Putative antennal carrier protein TOL-2	An. sinensis, 73%	4	Chemosensory/ Signal P: 1–19
	W5J836	Takeout	*An*. *darling*, 17%	3	Chemosensory/ Signal P: 1–19
**11/ 1.5**	B8RJ80	5'-AMP-activated protein kinase, alpha-2	*Culex tarsalis*, 30%	5	Phosphorylation
	B0WVM0	Beclin-1	*Culex quinquefasciatus*, 12%	6	Autophagy
	A0A023EM51	Putative heme oxygenase 1	*Ae*. *albopictus*, 20%	8	Heme oxidation
	W5JD00	26S proteasome regulatory subunitS3	*An*. *darling*, 15%	9	Proteolytic
	Q7QKL3	AGAP003249-PA	*An*. *gambiae*, 11%	8	Serine protease/ Signal P: 1–30
	A0A023EFV7	Putative galactose-specific c-type lectin	*Ae*. *albopictus*, 34%	4	Immune/ Signal P: 1–22
	B0WQX3	Aldehyde dehydrogenase	*Culex quinquefasciatus*, 10%	5	Oxidation reduction
	Q8I8P8	OBP39	*An*. *gambiae*, 14%	5	Chemosensory

**Table 3:** Annotated down regulated salivary proteins in *An*. *culicifacies* upon blood feeding.

Match ID/ Fold decrease	Accession	Protein	Organism, % sequence coverage	Peptide matches	Function/ Signal peptide if any
**0/0.17**	B0WIV8	Putative uncharacterized protein	*Culex quinquefasciatus*, 22%	7	Caspase recruitment domain
	A0A084VT63	Isocitrate dehydrogenase	*An*. *sinensis*, 29%	12	Oxidation reduction
**3/0.38**	A0A023EUU0	Putative juvenile hormone epoxide hydrolase ii	*Ae*. *albopictus*, 22%	8	Physiological change/ Signal P: 1–19
	Q6TRY1	Putative salivary OBP 2	*Culex quinquefasciatus*, 27%	2	Signal P: 1–20
	A0A023EVV5	Putative pftaire-interacting factor 1a	*Ae*. *albopictus*, 23%	14	Unknown
**12/0.37**	B0W7K8	Chemosensory protein 1	*Culex quinquefasciatus*, 71%	8	OBP 10
**14/0.42**	A0A023EFB5	Putative 11 kDa salivary protein	*Ae*. *albopictus*, 60%	4	Magnesium transport/ Signal P: 1–19
**27/0.34**	W5JJH0	Sphingosine phosphate lyase	*An*. *darling*, 13%	8	Apoptosis regulation
	O17491	Iron regulatory protein	*An*. *gambiae*, 17%	5	Iron homeostasis

**Fig 8:**
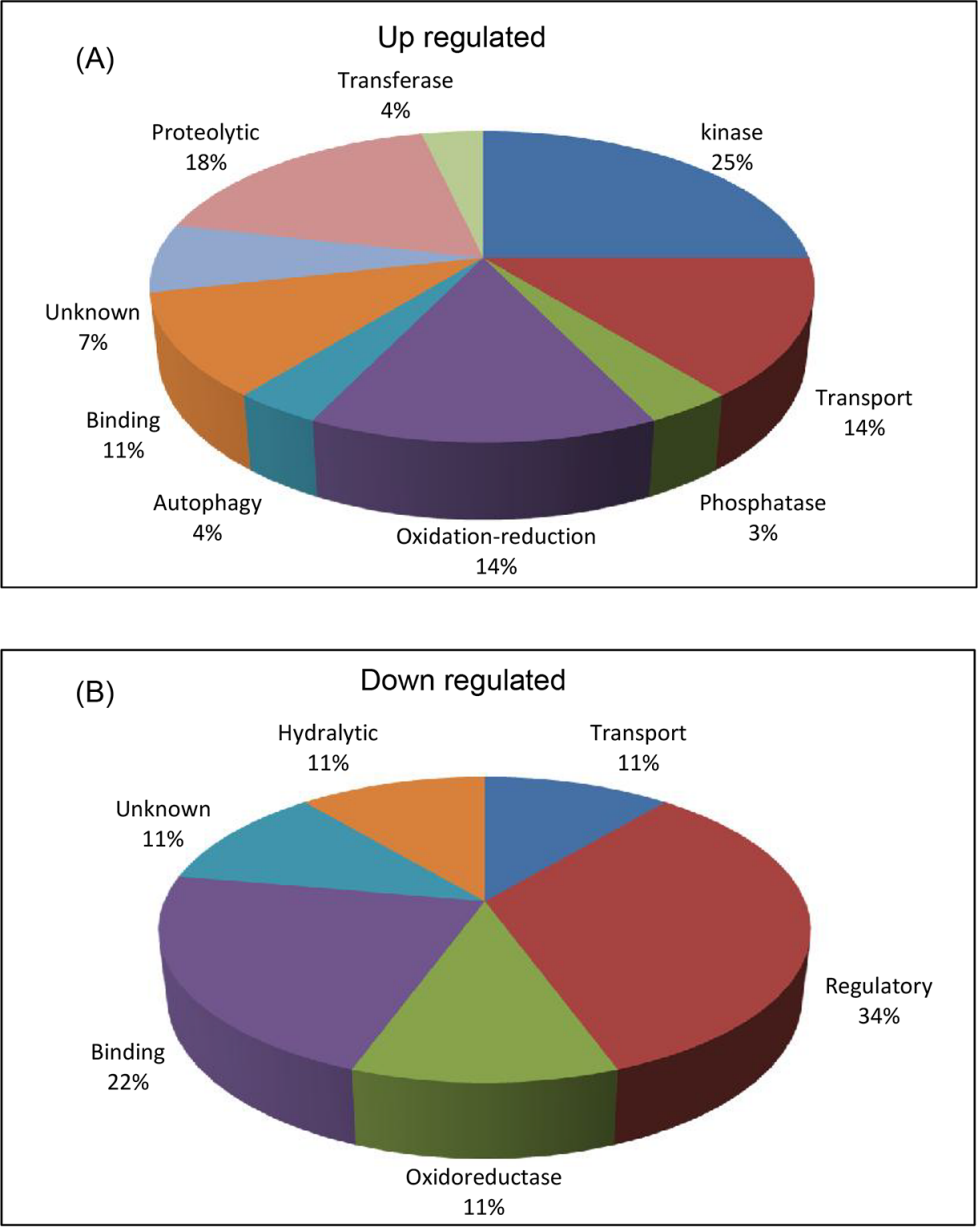
Molecular functions of differentially regulated salivary gland proteins analyzed using gene ontology tool. (A) GO function of identified up regulated proteins. (B) GO function of identified down regulated proteins.

Among up regulated proteins, majority of the proteins fell into categories of kinase function (25%) followed by proteolytic function (18%). These kinase proteins, serpins, signal transduction proteins, CLIP B protein, putative secreted proteins and 30kDa salivary antigen protein were found to be expressed 2 fold higher after blood meal as compared to sugar fed mosquitoes. Further, oxidoreductive proteins like glucose dehydrogenase, aldehyde dehydrogenase and detoxifying protein like cytochrome 450 were found to be 1.5 fold higher expressed in blood fed salivary glands than sugar fed. The down regulated proteins were mostly found to be having physiological functions (like chemosensory protein, odorant bindng protein etc) and regulatory functions namely apoptosis regulation, iron regulatory etc. Details of the molecular functions of up regulated and down regulated proteins in BF mosquitoes are shown in a pie chart (Fig 8A and 8B).

## Discussion

Salivary glands of mosquitoes are important organs and are the first target during the process of parasite invasion, maturation and subsequent transmission to the human host. Because of the direct contact of the salivary glands with the human host during biting and release of antiplatelet aggregation components and anti-inflammatory proteins to facilitate blood feeding, their importance in studying host, vector and parasite interactions is well known. Various studies have reported altered salivary proteins after blood meals using 1-DE and 2-DE in different mosquito species however few transcriptomic and no proteomic studies have been carried out in the salivary glands of *An*. *culicifacies* species and also genome sequence is unknown till date hence, in the current study, as a first step proteomic studies were carried out using 1-DE combined with LC-MS/MS and secondly, the differential expression and annotations of SF and BF compositions were compared using 2-DE coupled with MALDI-TOF mass spectrometry in salivary gland of *An*. *culicifacies*.

Our findings by both in-solution and in-gel approach identified a total of 106 putative salivary proteins of *An*. *culicifacies* and a baseline catalogue was prepared. Significant identified proteins in the catalogue included the members of D7 short form secreted proteins i.e. D7 r1, D7r2 and D7r4. These D7 family proteins are well described salivary gland proteins unique to dipterans and are distant relatives of the odorant binding protein superfamily [25–27] which actually bind host biogenic amines to antagonize vasoconstriction and platelet aggregating [25] and facilitate the blood feeding and potentially parasite ingestion from the vertebrate host. These proteins were also reported earlier in *Aedes aegypti* and *An*. *stephensi* using transcriptomic and proteomic approach [11, 13, 28]. Our results, together with other earlier studies in *Aedes aegypti* and *An*. *stephensi* indicate that this family of proteins are involved in hematophagy and potentially in parasite transmission during blood meal.

Salivary gland specific antigen 5 (Ag5) family proteins are extracellular proteins of ubiquitous family and are known to act as functional allergens in insects [29]. Another salivary gland specific family of enzymes reported in the insects and ticks are antihemostatic protein i.e. apyrases that act as vasodilator. During blood feeding nucleotidase activity of apyrase prevents ADP-induced platelet aggregation in the host and thereby helps in hematophagy [30]. Moreover the end product generated in the process i.e. AMP gets converted in to adenosine which acts as a powerful anti-inflammatory substance [31]. In addition, another enzyme salivary maltase was also identified which helps in the digestion of sugar meal [32]. Protein AGAP009642-PA with prohibitin domain was also identified in the profile. In insects like Drosophila and silkworm prohibitin is known to have role in normal development [33]. In a recent study, prohibitin was reported to act as a virus receptor during dengue transmission. It interacts with dengue E protein and is associated with dengue serotype 2 infection in *Aedes aegypti* and *Aedes albopictus* [34]. Various proteins involved in detoxification of xenobiotics and antioxidant were observed like glutathione S transferase (GST), theta and GST S1-2, peroxiredoxin 4, catalase, thioredoxine and dihydrolipoyl dehydrogenase. Results from the proteomic approach used in this study confirmed that *An*. *culicifacies* also possess anti-platelet aggregation (apyrase and anti-platelet protein) and anti-inflammatory (D7 and D7 related) proteins that may facilitate in blood feeding process. A detailed catalogue of these related proteins is presented in Table 1.

In our efforts to identify differentially expressed and annotated proteins in response to blood feeding, we have compared salivary gland proteins of sugar fed (SF) *An*. *culicifacies* species with blood fed (BF) mosquitoes by 2-DE which was combined with MALDI TOF to enlighten the important roles of salivary proteins in hematophagy. Interestingly, among the 29 upregulated proteins large array of phosphorylating /kinase enzymes namely ceramide kinase, serine/threonine kinase, calcium/calmodulin-dependent protein kinase type 1 etc were identified in *An*. *culicifacies* that are mainly involved in the function of autophagy and its regulation. It is known that autophagy process in response to various cellular stresses like starvation, pathogen infection etc. get upregulated and hence protect organism against cellular distress [35]. Various authors have demonstrated that these identified kinase enzymes like ceramide kinase [36], serine/threonine kinase and calcium/calmodulin-dependent protein kinase type 1 [37] etc. generates several proapoptotic signals that induce autophagy [38]. Therefore, our observations indicate that these identified proteins in salivary glands of blood fed mosquitoes might play a role as a survival factor against unwanted microorganism or pathogen and external stress enhanced during blood feeding. The role of autophagy was also implicated in epithelium protection against the products of blood digestion in insect as mentioned earlier by Rost-Roszkowskan et al [39]. In addition, Beclin-1/autophagy-related protein 6 was also identified to be up regulated in our studies that might play a central role in autophagy as suggested by earlier studies in Drosophila by Sharavage et al [40]. The phosphorylation of beclin-1 promotes disassociation of beclin 2-beclin 1 complex which activates autophagy [35, 41]. In a recent observations, the transcriptomics studies on *An*. *culicifacies* salivary glands also reports the upregulation of autophagy related proteins in response to blood feeding [42].

Oxysterol binding protein (OSBP) is known to be involved in signal transduction pathways and cellular lipid metabolism [43]. A study in *Aedes agypti* reported the up regulation of OSBP transcription after a blood meal [44,45]. In *Anopheles culicifacies*, OSBP protein was over expressed in blood fed confirming that OSBP plays a role in blood feeding. Up regulation of heme/iron assimilating proteins i.e. heme oxygenase enzyme 1 (HO1) and ferritin suggested that blood meal is responsible for iron overload [46]. It is known that autophagy process in response to various cellular stresses like starvation, pathogen infection etc. get upregulated and hence protect organism against cellular distress [47–48]. Caspases are cysteine proteases involved in the initiation and execution of apoptosis [49]. Elevated C-type lectin protein with carbohydrate recognition domain functions as receptor in pathogen recognition and play important role in insect immune response [50]. Enzymes related to energy metabolism i.e. aldehyde dehydrogenase and glucose dehydrogenase was also over expressed post blood meal. It has been reported that aldehyde dehydrogenase enzyme play a critical role in regulation of juvenile hormone (JH) synthesis in blood fed mosquitoes [51]. In adult female mosquitoes, JH controls the reproductive maturation [52] and induces the vitellogenesis [53]. Other over expressed proteins like antennal carrier protein TOL-2 (JH binding domain), takeout protein (JH binding domain) and odorant-binding proteins (OBP39) have role in regulation of blood feeding behaviours [54–55]. Reduced blood feeding after knockdown of takeout RNA in vivo in *An*. *gambiae* had been reported [56].

Over expression of 30 kDa salivary antigen family protein in *An*. *culicifacies* also correlates with the earlier studies in *Aedes aegypti* [57] and may provide valuable information on immune responses. The function of these 30 kDa salivary allergens remains unknown so far however; aegyptin a member of this family has been reported to inhibit collagen-induced human platelet aggregation [58].

Further, our findings showed the under expression of Juvenile hormone epoxide hydrolase (JHEH) II post blood meal. JHEHs are family of enzymes involved in irreversible degradation of juvenile hormones and have organ specific regulation [59]. Decreased expression of kreb cycle enzymes i.e. isocitrate dehydrogenase and Aconitase/IRP also correlates to the earlier publication in mosquitoes *An*. *gambiae* [27] and *Aedes* [57] respectively. However the importance of these down regulated enzymes associated with ATP synthesis and proteins that utilize ATP and their biological meaning related to blood feeding yet to be determined. It is also reported that these enzymes of kreb cycle were decreased after 24 hrs of blood meal [27].Down regulation of OBP 10 and OBP 2 identified in *An*. *culicifacies* justified the earlier publications by Wasinpiyamongkol et al that reported same depletion of OBP transcripts in SG of *Aedes aegypti* [57]. OBP’s are actually important for sensing however it was reported that after taking blood meal further OBP are not required until next blood meal and as the mosquito transitions to oviposition behavior [60].

Almost all the differentially expressed proteins in blood fed mosquito salivary glands seem to have key role in successful blood feeding. Blood feeding in mosquitoes depends on a number of factors namely host platelet activation, aggregation and coagulation and immune systems [6]. However, more studies are needed to further investigate the functions of novel and annotated salivary proteins to examine their functional role in mosquito feeding and host probing behaviour. Such studies using RNAi gene silencing assays on salivary gland transcribed genes may provide explanations on the secretion mechanisms of the mosquitos’ salivary glands. Since no such information is available in *An*. *culicifacies* mosquitoes such studies combined with this proteomic dataset will help to identify and characterise role of specific proteins whether in blood feeding or not because proteomic annotations does not clearly conclude their functional role.

## Conclusion

To our knowledge, this study presents the first proteomic baseline map and cataloging of the salivary glands of sugar fed female *An*. *culicifacies* with detailed putative functional annotation of all the identified proteins. The differences in the proteins suggest a plausible role in facilitating blood feeding but may also be implicated in the transmission of malaria parasites. Identification of novel expressed proteins and up and down regulated annotated proteins in blood fed mosquitoes using differential 2 DE method particularly several proteins related to autophagy like beclin-1, phosphorylating related proteins, heme oxygenase 1, ferritin, apoptotic, coagulation and immunity related proteins and proteins involved in regulation of blood feeding behavior and juvenile hormone may relate to the functions in the hematophagy. These correlations however, do not provide direct proof in enhancing of the blood feeding behavior. Understanding of these identified differentially regulated proteins after blood meal that may directly or indirectly be associated with development and egg laying capacity will provide insights into the physiological changes associated with feeding behavior, parasite transmission during blood feeding and may open the way for the development of novel malaria blocking strategies.
